# Supplementary material for: Surface-Enhanced Raman Spectroscopy Combined with Multivariate Analysis for Fingerprinting Clinically Similar Fibromyalgia and Long COVID Syndromes
Source: Biomedicines. 2024 Jun 28;12(7):1447. doi: 10.3390/biomedicines12071447 (PMC11275161; doi:10.3390/biomedicines12071447)
Supplement: Supplementary file 1 [file biomedicines-12-01447-s001.zip › biomedicines-3049085-supplementary.pdf]

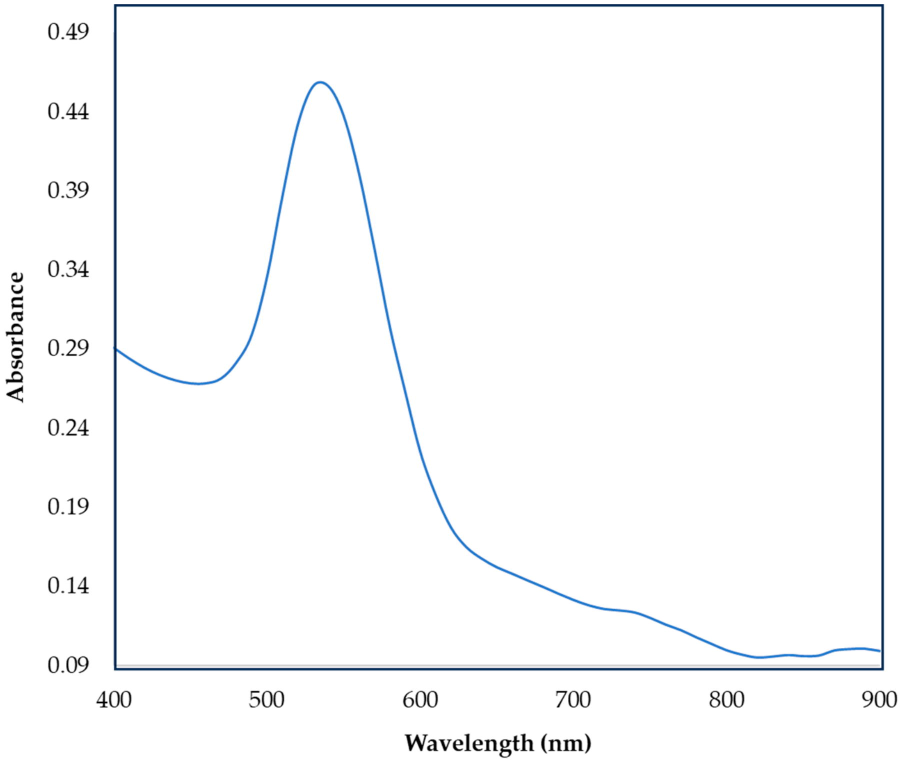

Figure S1. Average UV-visible spectra of AuNPs with average maximum absorbance at 540nm.

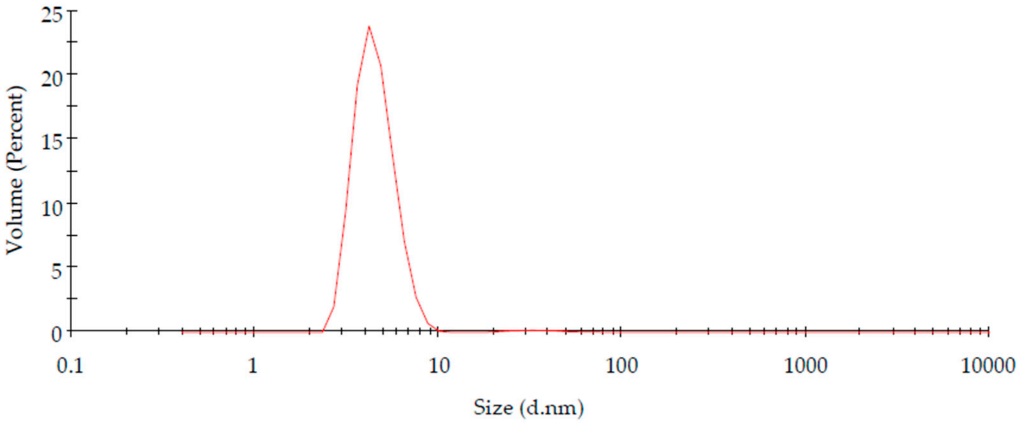

Figure S2. AuNPs (water and acetonitrile, 1:1) size distribution.

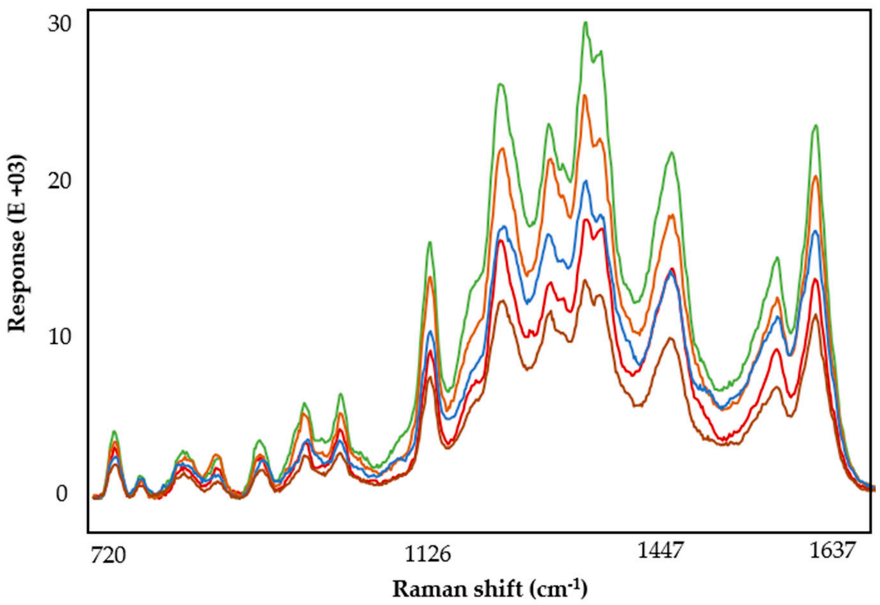

Figure S3. VAMS measurements illustrating spectral pattern consistency in FM sample regardless of signal intensity.

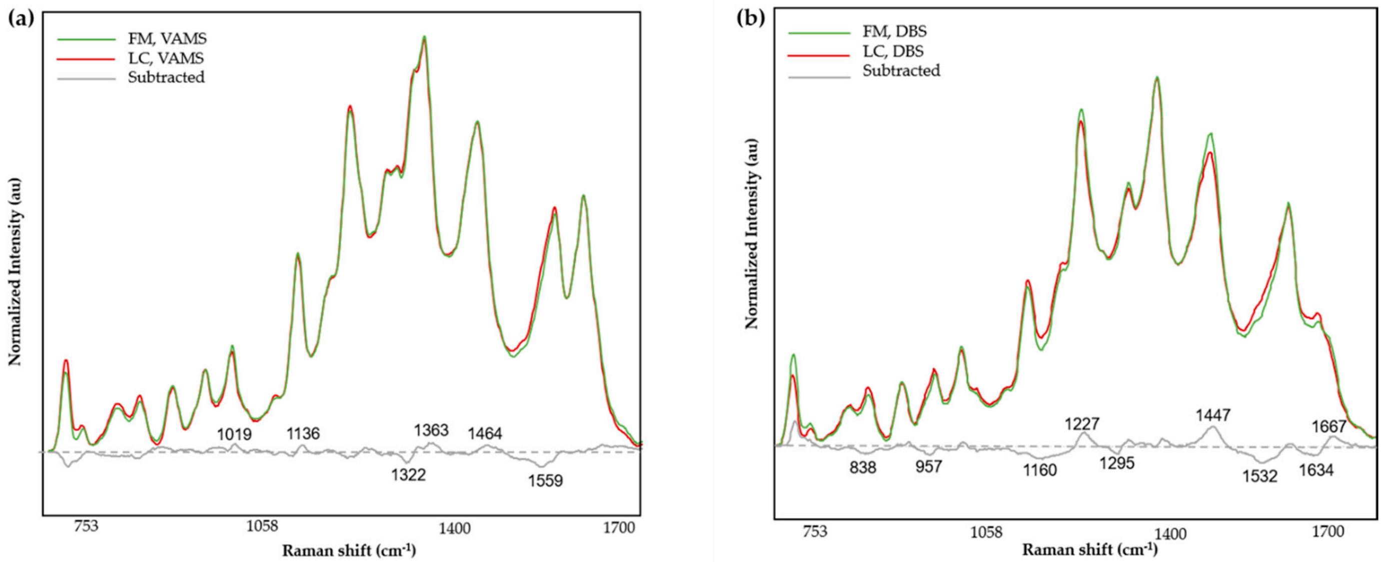

Figure S4. The subtracted mean spectra of LC from FM group, obtained from, **(a)** VAMS tips and **(b)** DBS cards.

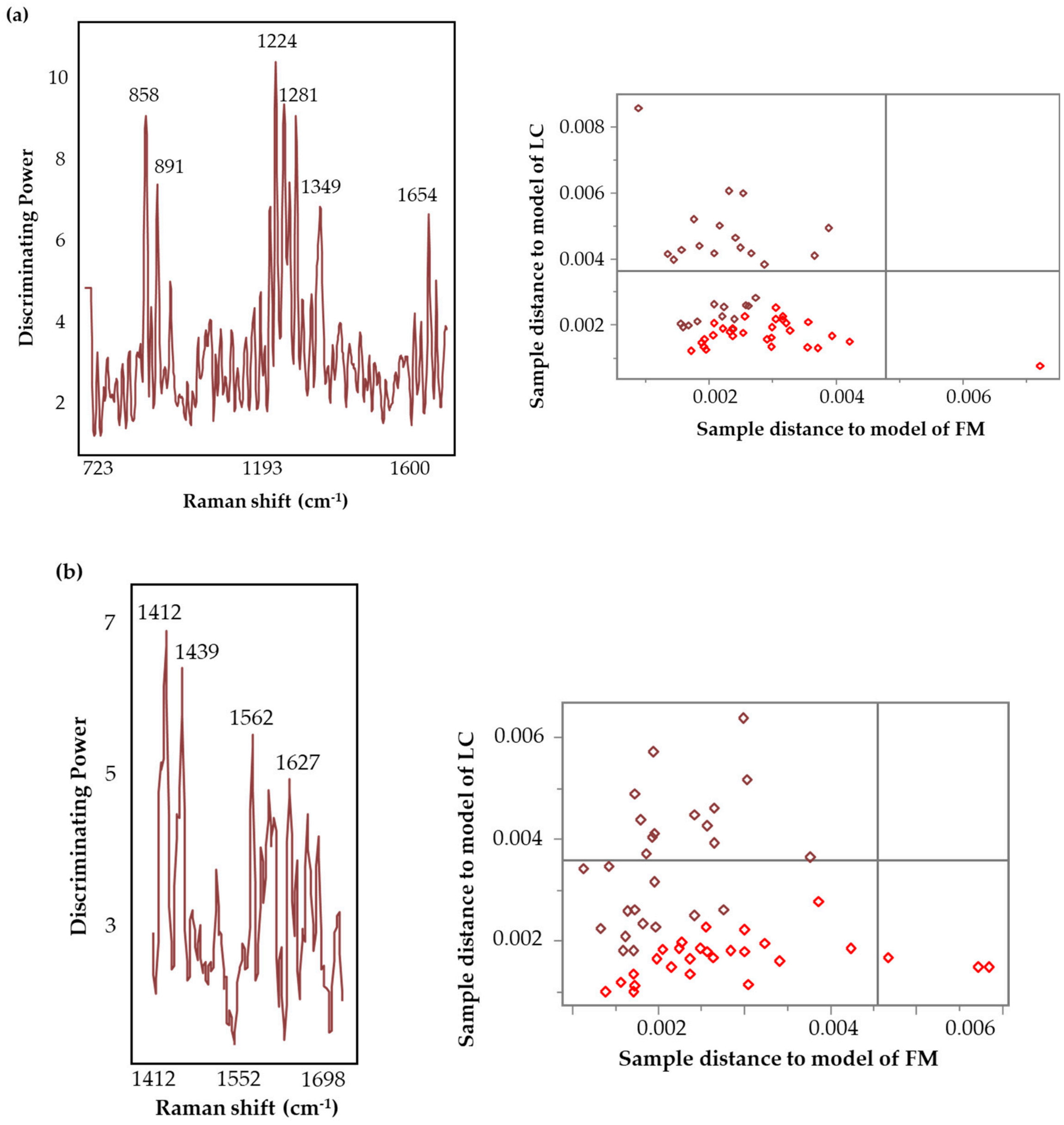

Figure S5. Discrimination power and Coomans plots (brown squares denote FM and red squares represent LC samples) of the SIMCA model for the full region (700-1700 cm^{-1}) **(a)** and the amide region (1400-1700 cm^{-1}) **(b)**, for the classification of FM and LC samples collected using VAMS tips.

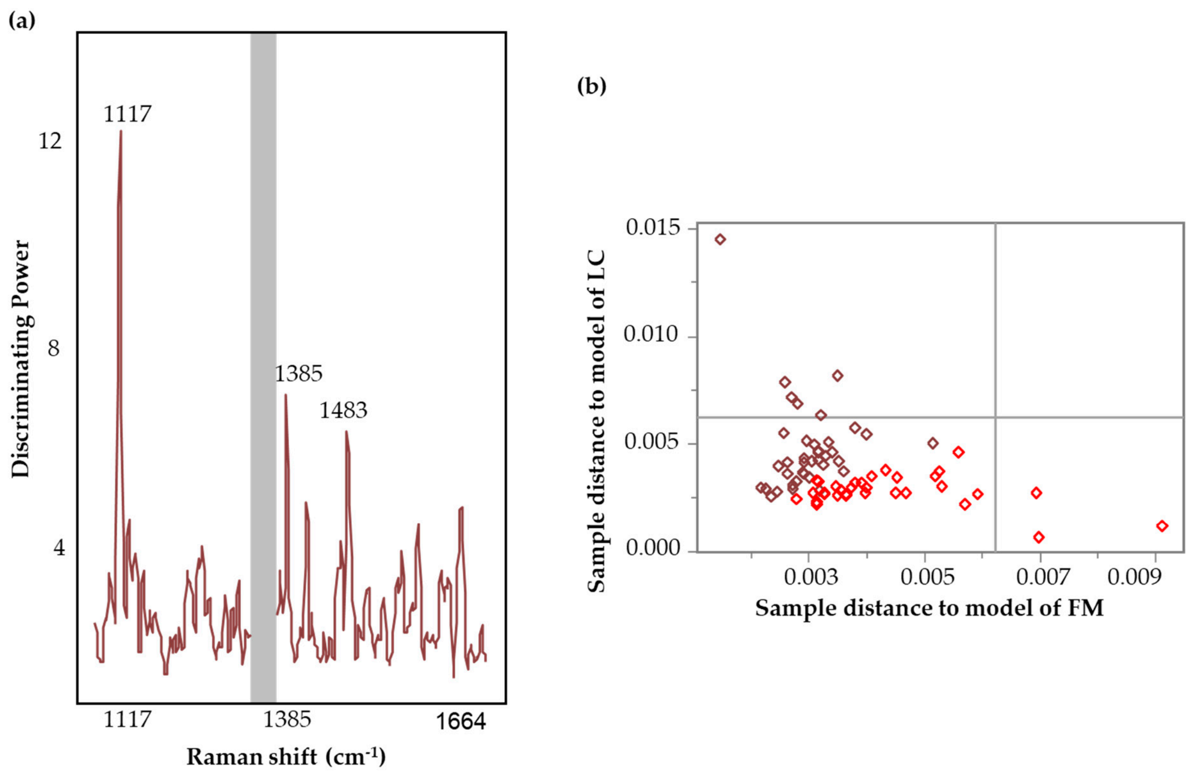

Figure S6. Discrimination power **(a)** and Coomans plots **(b)** (brown squares denote FM and red squares represent LC samples) of the SIMCA model for the central region (1100-1330 cm^{-1} and 1400-1700 cm^{-1}), for the classification of FM and LC samples collected on DBS cards.
